# Supplementary material for: Modeled Early Longitudinal PSA Kinetics Prognostic Value in Rising PSA Prostate Cancer Patients after Local Therapy Treated with ADT +/− Docetaxel
Source: Cancers (Basel). 2022 Feb 5;14(3):815. doi: 10.3390/cancers14030815 (PMC8834031; doi:10.3390/cancers14030815)

Supplementary data:

Supplementary Table S1: Baseline characteristics of the selected and excluded patients. Abbreviations: ADT, Androgen-Deprivation Therapy; DT, Doubling Time; IQR, InterQuartile Range; PSA, prostate-specific antigen. mo: months.

Characteristics	Selected patients (N=177)		Excluded patients (N=73)	
	ADT + Docetaxel	ADT alone	ADT + Docetaxel	ADT alone
Patients, No. (%)	94 (53.1)	83 (46.9)	31 (42.5)	42 (57.5)
Age, median (IQR), y	64 (58-70)	66 (61-71)	64 (58-70)	64 (59-69)
Stage T3/T4, No. (%)	55 (31.1)	46 (26.0)	18 (24.7)	28 (38.4)
Prior RP, No. (%)	64 (36.2)	57 (32.2)	26 (35.6)	33 (45.2)
Prior RT, No. (%)	30 (16.9)	26 (14.7)	5 (6.8)	9 (12.3)
**Baselines measurements, median (IQR)**				
PSA levels, ng/mL	3.3 (0.4-6.2)	3.2 (0.1-6.3)	1.5 (0.1-3.1)	1.6 (0-3.2)
PSA-DT, mo.	5.9 (3-8.8)	6.1 (2.8-9.4)	5.8 (4.7-6.9)	5.2 (3.7-6.7)
PSA velocity, ng/mL/year	1.4 (0.5-2.3)	1.4 (0.7-2.1)	1.4 (1.1-1.7)	1.6 (1.1-2.1)
**High-risk factors at inclusion, No. (%)**				
Gleason score ≥ 8	32 (18.1)	25 (14.1)	9 (12.3)	11 (15.1)
PSA-DT ≤ 6 mo.	49 (27.7)	40 (22.6)	18 (24.7)	27 (40.0)
PSA velocity > 0.75 ng/mL/year	78 (44.1)	64 (36.2)	28 (38.4)	37 (50.1)

Supplementary Table S2: Final parameter estimates. IIV: Inter-individual Variability. ** Inter-Individual coefficient of variation (CV) calculated as square root(variance of random effect) × 100. RSE: Relative standard Error. BSV: Between-Subject Variability. AU: Arbitrary Unit. RSE scale: standard deviation.

Parameters	Population estimates		IIV** (%CV)		BSV (%)	Shrinkage (%)
	Estimate	RSE (%)	Estimate	RSE (%)		
K (day^{-1})	2.130	9.691	0.064	11.659	25.259	97.265
***KPROD** (ng.ml^{-1}.day^{-1})*	**0.002**	**8.794**	**4.260**	**5.593**	**206.398**	**18.114**
EC50 (AU)	0.357	5.311	0.145	4.559	38.079	97.354
PSA0E (ng.mL^{-1})	4.220	6.704	1.680	5.158	129.615	13.277
***KELIM** (day^{-1})*	**0.081**	**5.302**	**0.124**	**6.111**	**35.214**	**26.883**

Supplementary Figure S1: Model validation. A. GOF plots for Observed PSA assays vs. Individual Predictions of PSA.B. Normalized Prediction Distribution Errors distribution (NPDE). C. Visual Predictive Check (VPC). Top figure are for data above limit of quantification and bottom figure represents the probability of getting an assay lower than the limit of quantification. Red lines represent the median (solid line), and the 10th and 90th percentiles (dashed lines) of the observed PSA values. Blue areas represent the 95% confidence intervals of PSA simulations.

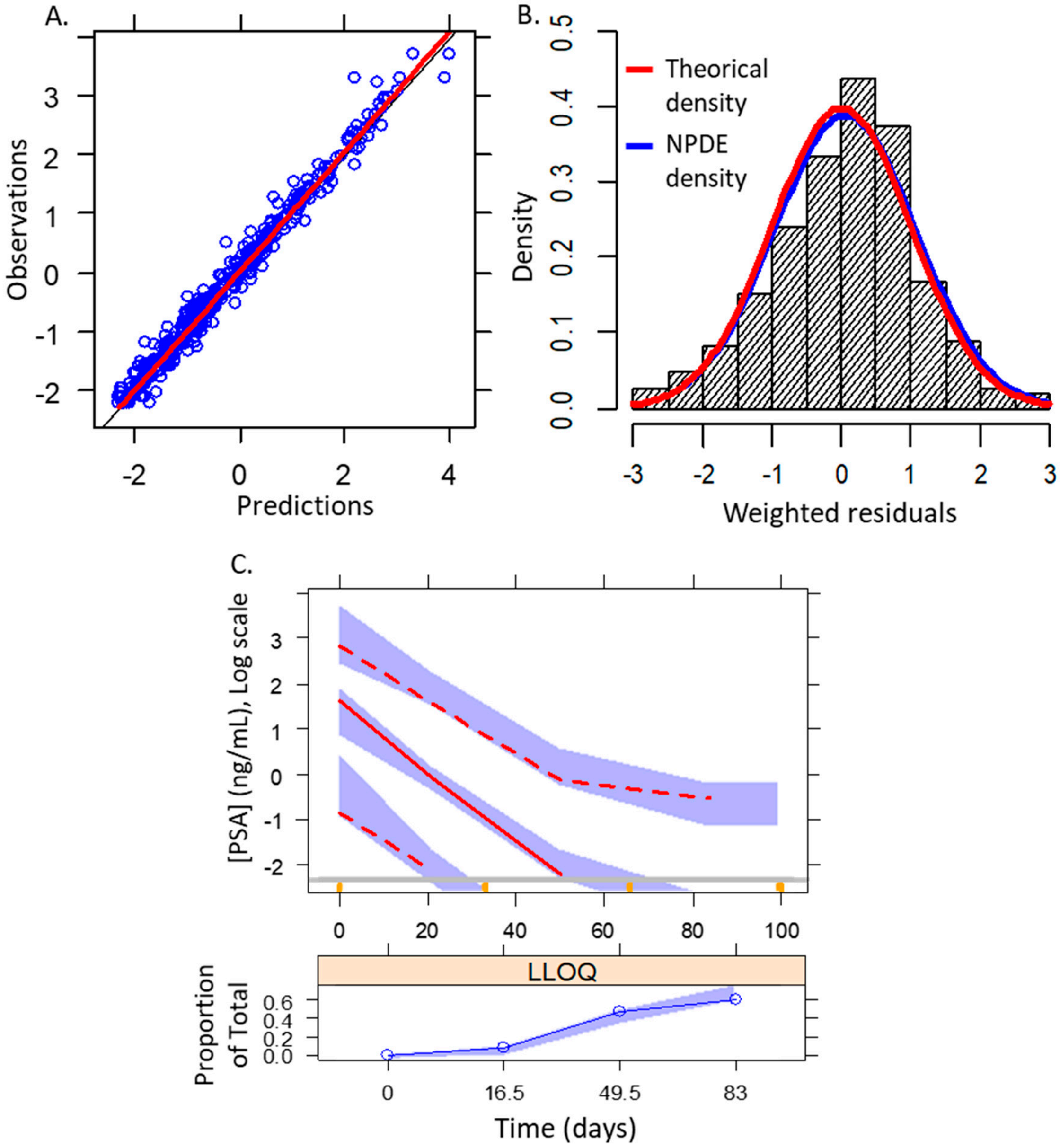

Supplement: Supplementary file 1 [file cancers-14-00815-s001.zip › cancers-1565868-supplementary.pdf]
